# The Effect of Serotonin-Targeting Antidepressants on Neurogenesis and Neuronal Maturation of the Hippocampus Mediated via 5-HT1A and 5-HT4 Receptors

**DOI:** 10.3389/fncel.2017.00142

**Published:** 2017-05-16

**Authors:** Eri Segi-Nishida

**Affiliations:** Department of Biological Science and Technology, Faculty of Industrial Science and Technology, Tokyo University of ScienceTokyo, Japan

**Keywords:** antidepressant, neurogenesis, 5-HT4 receptor, hippocampus, maturation, granule cell

## Abstract

Antidepressant drugs such as selective serotonin reuptake inhibitors (SSRIs) specifically increase serotonin (5-HT) levels in the synaptic cleft and are widely used to treat mood and anxiety disorders. There are 14 established subtypes of 5-HT receptors in rodents, each of which has regionally different expression patterns. Many preclinical studies have suggested that the hippocampus, which contains abundant 5-HT1A and 5-HT4 receptor subtypes in the dentate gyrus (DG), is critically involved in the mechanisms of action of antidepressants. This review article will analyze studies demonstrating regulation of hippocampal functions and hippocampus-dependent behaviors by SSRIs and similar serotonergic agents. Multiple studies indicate that 5-HT1A and 5-HT4 receptor signaling in the DG contributes to SSRI-mediated promotion of neurogenesis and increased neurotrophic factors expression. Chronic SSRI treatment causes functions and phenotypes of mature granule cells (GCs) to revert to immature-like phenotypes defined as a “dematured” state in the DG, and to increase monoamine reactivity at the dentate-to-CA3 synapses, via 5-HT4 receptor signaling. Behavioral studies demonstrate that the 5-HT1A receptors on mature GCs are critical for expression of antidepressant effects in the forced swim test and in novelty suppressed feeding; such studies also note that 5-HT4 receptors mediate neurogenesis-dependent antidepressant activity in, for example, novelty-suppressed feeding. Despite their limitations, the collective results of these studies describe a potential new mechanism of action, in which 5-HT1A and 5-HT4 receptor signaling, either independently or cooperatively, modulates the function of the hippocampal DG at multiple levels, any of which could play a critical role in the antidepressant actions of 5-HT-enhancing drugs.

## Introduction

Serotonin (5-hydroxytryptamine, 5-HT) is a monoamine neurotransmitter that plays important roles in physiological and pathophysiological functions in the brain. Serotonergic neurons from the dorsal and median raphe project throughout the brain, including sending afferents to the hippocampus, one of the stress-sensitive limbic structures implicated in the pathophysiology of mood and anxiety disorders (Pittenger and Duman, 2008; Kheirbek et al., 2012). Drugs such as selective serotonin reuptake inhibitors (SSRIs) specifically increase 5-HT levels in the synaptic cleft and are widely used to treat mood and anxiety disorders. Preclinical studies demonstrate that stress leads to the atrophy and loss of hippocampal neurons (Pittenger and Duman, 2008; McEwen et al., 2016). In addition, many, but not all, neuroimaging studies report that the hippocampal volume is relatively small in patients with depression (MacQueen and Frodl, 2011). Conversely, chronic SSRI treatment increases the dendritic spine density of neurons in the CA pyramidal cell subfields and stimulates adult neurogenesis in the dentate gyrus (DG; Malberg et al., 2000; Santarelli et al., 2003; Bessa et al., 2009). Recent reports also indicate that chronic SSRI treatment significantly changes the physiological and functional properties of mature granule cells (GCs), the primary cell type of the DG (Kobayashi et al., 2010). These findings demonstrate that enhancing serotonergic activity with antidepressants modulates hippocampal function. Therefore, newer research has been focused on the signaling pathways activated by SSRIs in the hippocampus.

At least 14 different 5-HT receptor types and subtypes have been identified (Hoyer et al., 2002). Among them, 5-HT1A, 5-HT1B, 5-HT2B, 5-HT4 and 5-HT7 receptors are expressed in the DG of the hippocampus (Pompeiano et al., 1992; Grossman et al., 1993; Bruinvels et al., 1994; Vilaró et al., 1996; Klempin et al., 2010; Tanaka et al., 2012; Diaz et al., 2013). Although there are some cell-type-related exceptions, the 5-HT1A and the 5-HT1B receptors generally couple to Gi/o proteins, the 5-HT2B receptor couples to Gq/11 proteins, and the 5-HT4 and the 5-HT7 receptors couple to Gs proteins (Bockaert et al., 2008; Polter and Li, 2010).

This review article discusses studies demonstrating multiple forms of regulation of hippocampal functions mediated by SSRIs and serotonergic agents. A recent review has addressed the general roles of 5-HT1A and 5-HT4 receptors in the actions of antidepressants (Samuels et al., 2016), but this mini-review article focuses specifically on the contribution of 5-HT1A and 5-HT4 receptor activity to functional modulation of GCs in the DG. It will also briefly discuss the involvement of 5-HT1A and 5-HT4 receptors in hippocampus-dependent antidepressant and anxiolytic behavioral effects.

## Enhancement of Adult Neurogenesis in the Hippocampus by SSRIs and Relative Involvement of 5-HT Receptor Subtypes in the DG

Hippocampal neurogenesis is a dynamic process that is controlled by various factors, including environmental stimuli. In the mammalian DG, new neurons are generated from neural stem cells and progenitors in the subgranular zone (SGZ) of the DG throughout life (Ming and Song, 2005). Hippocampal neurogenesis is enhanced by active environmental stimulation such as learning, exercise and rich environmental conditions. Conversely, stress exposure or aversive stimulation suppresses this process. Preclinical studies demonstrate that exposure to chronic stress reduces proliferation of neuronal progenitors in the DG, resulting in decreased neurogenesis (Fuchs et al., 2006). Many, but not all, clinical studies also show reductions in hippocampal volume under depression and stress conditions (MacQueen and Frodl, 2011); together, these findings suggest that the reduction of neurogenesis by stress may be one of the cellular mechanisms underlying hippocampal structural changes in patients with depression.

Studies performed over the past 20 years provide evidence that SSRI-mediated acceleration of neurogenesis is one of the cellular mechanisms underlying antidepressant efficacy. Chronic administration of SSRIs such as fluoxetine facilitates each stage of neurogenesis, including progenitor proliferation, survival, and the early phase of maturation (Encinas et al., 2006; Wang et al., 2008). Fluoxetine administration also restores neurogenesis decreased by stress exposure. Inescapable electrical shock stress reduces cell proliferation in the adult rat hippocampus in a manner reversed by chronic fluoxetine treatment (Malberg and Duman, 2003). Chronic corticosterone treatment in mice, a model of anxiety and depression, also reduces proliferation of progenitor cells. In this model, the effect of fluoxetine at each stage of neurogenesis including proliferation, differentiation, and survival is more pronounced than that in control mice (David et al., 2009), suggesting that corticosterone treatment increases the sensitivity of hippocampal neurogenesis to fluoxetine. In humans, the number of neuronal progenitors in the DG of SSRI-treated patients with major depressive disorder is higher than that in untreated patients (Boldrini et al., 2009). Consistent with this report, the number of mature GCs in the DG of SSRI-treated patients with major depressive disorder is also higher than that in untreated patients (Boldrini et al., 2013).

Multiple evidences have demonstrated that the 5-HT1A receptor in mature GCs contribute to the promotion of hippocampal neurogenesis by SSRIs. *In situ* hybridization studies demonstrate abundant 5-HT1A mRNA expression in mouse GCs (Pompeiano et al., 1992; Tanaka et al., 2012; Diaz et al., 2013). Pharmacological studies show that a 5-HT1A receptor agonist, 8-OH-DPAT, increased proliferation in the DG upon short-term administration in mice or rats (Banasr et al., 2004; Klempin et al., 2010; Arnold and Hagg, 2012; reviewed in Alenina and Klempin, 2015). Conversely, chronic treatment with 5-HT1A receptor antagonists (WAY100135 or NAN-190) decreases proliferation and survival of newborn cells in the DG in some studies, but not all (Radley and Jacobs, 2002; Zhang et al., 2016). Furthermore, germline 5-HT1A receptor knockout mice show lack of effects of the SSRIs on cell proliferation in the DG (Santarelli et al., 2003). However, since the 5-HT1A receptor is expressed not only in GCs as a heteroreceptor but also in serotonergic raphe neurons as an autoreceptor, it is unclear whether 5-HT1A signaling in GCs directly influences neurogenesis. Recently, the function of the 5-HT1A receptor in the hippocampal DG was examined using mice lacking the 5-HT1A receptor specific to GCs (Samuels et al., 2015). Fluoxetine-induced facilitation in cell proliferation and early neural maturation in the DG are attenuated in mice lacking GC-specific 5-HT1A receptor, demonstrating that postsynaptic 5-HT1A signaling in GCs is involved in hippocampal neurogenesis induced by fluoxetine.

Recent studies have also implicated that the 5-HT4 receptor signaling contributes to the promotion of hippocampal neurogenesis by SSRIs. Specific ligand binding and *in situ* hybridization studies demonstrate abundant 5-HT4 expression in mouse or rat DG (Grossman et al., 1993; Vilaró et al., 1996; Tanaka et al., 2012; Diaz et al., 2013; Imoto et al., 2015). Pharmacological studies demonstrate that the proliferative effect of a 5-HT4 agonist (RS67333) is observed in the rat DG following a short term administration protocol (Lucas et al., 2007; Pascual-Brazo et al., 2012). Chronic activation of the 5-HT4 receptor facilitates not only proliferation, but also maturation in newborn neurons, and chronic inhibition of 5-HT4 receptor partially blocks the neurogenic effect of chronic fluoxetine (Mendez-David et al., 2014). Another line of study also demonstrates that germline 5-HT4 receptor knockout mice of the C57BL/6J strain are resistant to the effects of fluoxetine on the proliferation of newborn cells and the number of immature neurons in the DG (Imoto et al., 2015). Since there is no report of GC-specific 5-HT4 receptor knockout mice, it is unknown whether 5-HT4 receptors act specifically in the GCs to contributes to neurogenesis therein. However, several evidences indicate that the 5-HT4 receptor activates the intracellular signaling of GCs. For example, the short-term stimulation of 5-HT4 receptors increases cAMP response element binding protein (CREB) activation and brain derived neurotrophic factor (BDNF) expression in the DG (Lucas et al., 2007; Pascual-Brazo et al., 2012). Thus, increased 5-HT4 activity in mature GCs may directly facilitate gene expression of neurotrophic factors in the DG, and contribute to the hippocampal neurogenesis. It is still possible that 5-HT4 receptors expressed in other brain regions could affect neurogenesis. For example, the 5-HT4 receptor in the prefrontal cortex is found to increase the activity of dorsal raphe serotonergic neurons (Lucas and Debonnel, 2002; Compan et al., 2004).

It is also important to note that serotonergic activity is critical for exercise-induced adult hippocampal neurogenesis (Klempin et al., 2013). It would be interesting to investigate the 5-HT receptor subtypes and mechanisms involved in exercise-induced neurogenesis and then to compare the results with those of SSRI-induced neurogenesis. Interestingly, neither the 5-HT1A receptor- nor the 5-HT4 receptor-deficient mice have changes in basal hippocampal neurogenesis (Santarelli et al., 2003; Imoto et al., 2015), suggesting that neither 5-HT1A nor the 5-HT4 activity is necessary for the maintenance of neurogenesis. Therefore, these signals may play an important role in regulating hippocampal activity in response to environmental and pharmacological stimuli.

## Ssri-Related Functional and Phenotypic Changes in Mature Granule Neurons and the Involvement of Specific 5-HT Receptor Subtypes

Mature GCs in the DG, which is positioned at the entrance of the hippocampal excitatory trisynaptic circuit, play a critical role in regulating hippocampal function. The majority of neurons in the adult DG are mature GCs. Electrophysiological studies show that GC mossy fibers regulate associative synaptic plasticity in the hippocampal CA3 region (Contractor et al., 2000; Lauri et al., 2001; Feng et al., 2003; Kobayashi and Poo, 2004). The abundant expression of 5-HT1A and 5-HT4 receptors in mature GCs suggests that serotonergic afferents regulate the function of the DG. However, it remains to be explained how sustained elevation of 5-HT levels in the synaptic cleft by chronic SSRI treatment affects the function and phenotypes of mature GCs.

**Figure 1:**
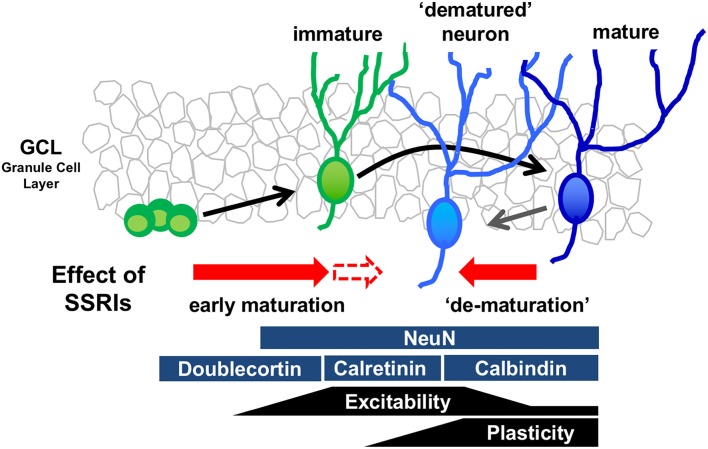
**Model of bidirectional effects of chronic serotonin (5-HT) reuptake inhibitors (SSRIs) exposure on the regulation of the state of maturation in the granule cells (GCs).** Developmental stages of GCs in the dentate gyrus (DG) can be identified by stage marker expression and neural function. Chronic SSRI administration may have bidirectional effects on differentiating immature neurons and mature neurons, depending on the state of differentiation and maturation.

The mature state of GCs is characterized by several distinct molecular and physiological features (Figure 1). Recent reports demonstrate that chronic treatment of SSRIs such as fluoxetine and paroxetine in adult mice changes several features of mature GCs, essentially transforming the cells into an immature-like phenotype defined here as a “dematured” state (Kobayashi et al., 2010, 2013). Electrophysiological analysis shows that fluoxetine-treated GCs are more easily excited by somatic current injection, and demonstarate markedly reduced synaptic facilitation in dentate-to-CA3 signal transmission, a condition characterizing the immature GC-to-CA3 synapse (Marchal and Mulle, 2004). In contrast, fluoxetine-treated GCs have unaltered input resistance and membrane time constant, indicating that fluoxetine did not induce drastic changes in the cell size or morphology in mature GCs (Kobayashi et al., 2010). Chronic fluoxetine also reduces expression of mature GC markers, such as calbindin and tryptophan-2,3-dioxygenase, which are normally highly expressed (Yamasaki et al., 2008). Several animal mutation-based models, including SNAP-25 mutant mice and Schnurri-2 knockout mice, possess “immature” cellular phenotypes in the DG (Ohira et al., 2013; Takao et al., 2013). A transcriptome analysis in the hippocampus shows that gene expression patterns are significantly similar between SNAP-25 mutant mice and fluoxetine-treated mice (Ohira et al., 2013), suggesting that fluoxetine-treated mice and SNAP-25 mutant mice demonstrate a similar immature GC phenotype. Many studies have shown that chronic fluoxetine treatment accelerates the early maturation process, including the corresponding increase in dendritic complexity (Wang et al., 2008) and decrease in the proportion of expression of the immature GC marker, calretinin, to newborn neurons (Klempin et al., 2010). These data suggest that chronic fluoxetine may have different effects on immature differentiating neurons and mature neurons, depending on the state of differentiation and maturation (Figure 1).

In a mouse model of depression and anxiety generated by chronic corticosterone treatment, changes in the maturation phenotypes of GCs can be induced at serum concentrations of fluoxetine that are close to those observed in patients during fluoxetine treatment (Kobayashi et al., 2013). Furthermore, electroconvulsive stimulation in animals, which simulates electroconvulsive therapy (a highly effective and fast-acting treatment for depression) in people, rapidly induces dematuration changes in GCs (Imoto et al., 2017). Together, these data imply a degree of clinical relevance for these phenotypic changes in GCs. Indeed, they suggest that dematuration of neurons in the DG could be a common cellular basis for the therapeutic effects of pharmacological and non-pharmacological antidepressant treatments. It is possible that the alteration in maturation phenotypes in GCs following antidepressant treatment may restore cell function; that is, it may act as a “reset” button for GCs. 5-HT4 receptor activity contributes to fluoxetine-induced maturation phenotype changes in GCs in addition to facilitation of neurogenesis (Kobayashi et al., 2010). The fluoxetine-induced expression of BDNF in the DG is associated with dematuration (Imoto et al., 2015). These studies suggest that sustained elevation of 5-HT levels in the synaptic cleft by chronic fluoxetine changes the phenotypes of GCs via the 5-HT4 receptor, which may alter the transcriptome pattern of the GCs and subsequently contribute to an increase in neurogenesis in the dentate SGZ.

**Figure 2:**
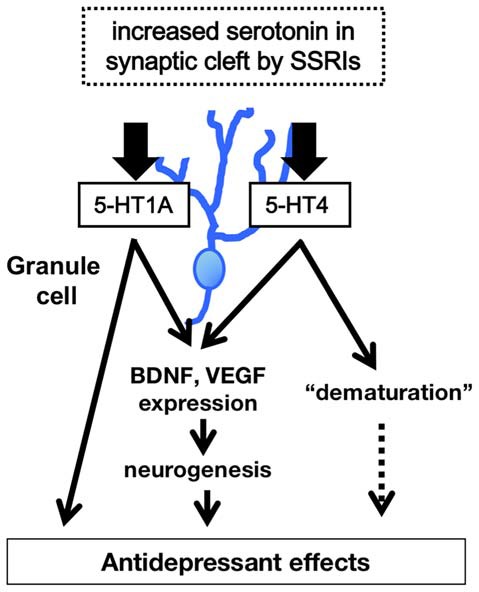
**Model of 5-HT1A and 5-HT4 receptor-mediated modulation of hippocampal function.** The higher level of 5-HT in synaptic cleft in the DG induces increase in expression of neurotrophic factors such as brain derived neurotrophic factor (BDNF), facilitation of hippocampal neurogenesis, and dematuration of GCs via the 5-HT1A and 5-HT4 receptors in the DG. The signals of 5-HT1A and 5-HT4 receptors in the DG contribute to several antidepressant and anxiolytic behaviors.

Recent studies also demonstrate that fluoxetine enhances monoamine reactivity at the synapses formed by GC-derived mossy fibers. 5-HT and dopamine potentiate mossy fiber-CA3 pyramidal cell synaptic transmission in mouse hippocampal slices (Kobayashi et al., 2010, 2012); chronic fluoxetine or paroxetine treatment induces a prominent increase in the magnitude of such potentiation. Synaptic potentiation by dopamine is mediated via D1-like receptors whose expression in the DG is upregulated by chronic fluoxetine, indicating their potential involvement in enhanced dopamine sensitivity mediated by fluoxetine. Synaptic potentiation by 5-HT is mediated via 5-HT4 receptors; however, chronic fluoxetine does not increase the expression of the 5-HT4 receptor in the DG, suggesting that the increase in 5-HT sensitivity is likely mediated by downstream intracellular signaling (e.g., second messenger signaling). Both 5-HT4 and D1-like receptors are cAMP-dependent (via Gs coupling). This enhanced monoaminergic modulation would greatly increase excitatory drive to the hippocampal circuit through the DG. Although the influence of 5-HT1A signaling on the function and phenotypes of mature GCs is unclear, studies using non-neuronal and neuronal cell lines indicate that 5-HT1A signal transduction is linked to not only the conventional Gi/o-mediated signaling pathway, but also to the mitogen-activated protein kinase (MAPK) and Akt signaling pathways. The latter pathways are thought to be associated with neurotrophic or growth factor expression in GCs (see description below, Samuels et al., 2015; Rojas and Fiedler, 2016). Taken together, functional and phenotypic changes by SSRIs in the mature GCs modulate hippocampal function, and this effect may contribute, in part, to their behavioral actions (Figure 2).

## Hippocampal-Dependent Behavioral Changes by SSRIs Mediated by Specific 5-HT Receptor Subtypes

Recent evidence suggests that the DG plays an important role in antidepressive and anxiolytic behavioral changes. Using optogenetic manipulation, increased ventral DG activity suppresses innate anxiety in mice (Kheirbek et al., 2013), while re-activating GCs in the DG that were previously active during a positive experience suppress depression-like behaviors (Ramirez et al., 2015). These findings indicate that functional regulation of the DG by SSRIs could contribute to their behavioral actions.

A recent study shows that the 5-HT1A receptor on mature GCs is critical for several antidepressant effects induced by chronic fluoxetine treatment (Samuels et al., 2015). Mice lacking GC-specific 5-HT1A receptors exhibit deficits in the antidepressant and anxiolytic effects of fluoxetine when tested with the forced swim test, novelty-suppressed feeding, and elevated plus maze. In contrast, 5-HT1A receptor missing from only young adult-born GCs produces normal fluoxetine-mediated responses, indicating the important role of the 5-HT1A signal in mature GCs. Fluoxetine-induced increases in BDNF and vascular endothelial growth factor (VEGF) expression levels in the DG are also attenuated in mice lacking GC-specific 5-HT1A receptors.

The 5-HT4 receptor is also thought to contribute to the behavioral effects of fluoxetine. In a model of anxiety and/or depression, antidepressant and anxiolytic effects caused by fluoxetine treatment are attenuated by administration of a 5-HT4 receptor antagonist; the same result is found in 5-HT4 receptor knockout mice (Mendez-David et al., 2014; Amigó et al., 2016). Since the 5-HT4 receptor antagonist suppresses the effect of fluoxetine on novelty-suppressed feeding, which is a neurogenesis-dependent antidepressant behavior, the 5-HT4 receptor-mediated increase of neurogenesis in the DG appears to be involved in the behavioral actions of fluoxetine. The functional and phenotypic changes in mature GCs due to 5-HT4 receptor activity may also have an influence on mature GC-dependent behaviors. Chronic fluoxetine treatment with high doses induces behavioral destabilization that is characterized by alternating between hypoactivity and hyperactivity within a few days; this behavioral destabilization by fluoxetine is tightly associated with fluoxetine-induced dematuration of the GCs and is attenuated in mice lacking 5-HT4 receptors (Kobayashi et al., 2011). Although behavioral destabilization may be caused by an increase in hippocampal excitability, it is unknown whether this behavioral change is involved in antidepressant activity. In a depression model in which hippocampal activity is suppressed, SSRI-induced functional and phenotypic changes of GC by the 5-HT4 receptor would hypothetically correct some of the hippocampally mediated, depression-like behaviors.

## Conclusions

In summary, a large body of evidence indicates that 5-HT signaling regulates hippocampal function and changes several antidepressant and anxiolytic behaviors through activity at both 5-HT1A and 5-HT4 receptors. It will be necessary to investigate how the 5-HT1A and the 5-HT4 receptors cooperate to activate downstream signals in the dentate GCs. In particular, identifying molecular changes that are induced by prolonged serotonin elevation is important for the development of new antidepressant drugs with a more rapid onset of action. Recent studies also indicate that chronic treatment with SSRIs not only promotes generation of new neurons, but also induces dematuration in existing GCs in the hippocampal DG, implicating that sustained elevation of serotonin levels in the synaptic cleft by chronic SSRIs may have bidirectional effects on GC maturation depending on the maturational stage of the cells. Although there are some limitations to these data, the results of these studies suggest that in addition to the 5-HT1A receptor, 5-HT4 receptor-mediated dematuration in mature GCs, as well as 5-HT4 receptor-mediated neurogenic effects, are responsible for the modulation of hippocampal function by SSRIs (Figure 2). Furthermore, these activities probably affect hippocampal-dependent behaviors and could play an important role in the actions of antidepressant treatment.

## Author Contributions

ES-N planned, organized and wrote the review article.

## Funding

This work was supported in part by MEXT KAKENHI (Grant Number 25460096, 17K08316, ES-N), grants from the Naito Foundation (ES-N), the foundation of Pharmaceutical Sciences (ES-N), and Astellas Foundation (ES-N).

## Conflict of Interest Statement

The author declares that the research was conducted in the absence of any commercial or financial relationships that could be construed as a potential conflict of interest.
